# Chemical and mechanical interfacial degradation in bifacial glass/glass and glass/transparent backsheet photovoltaic modules

**DOI:** 10.1002/pip.3602

**Published:** 2022-06-29

**Authors:** Laura Spinella, Soňa Uličná, Archana Sinha, Dana B. Sulas‐Kern, Michael Owen‐Bellini, Steve Johnston, Laura T. Schelhas

**Affiliations:** ^1^ National Renewable Energy Laboratory Golden CO USA; ^2^ SLAC National Accelerator Laboratory Menlo Park CA USA

**Keywords:** adhesion, bifacial, DuraMAT, EVA, glass–glass, ion migration, POE

## Abstract

Glass/glass (G/G) photovoltaic modules are quickly rising in popularity, but the durability of modern G/G packaging has not yet been established. In this work, we examine the interfacial degradation modes in G/G and glass/transparent backsheet modules under damp heat (DH) with and without system bias voltage, comparing emerging polyolefin elastomer (POE) and industry‐standard poly(ethylene‐co‐vinyl acetate) (EVA) encapsulants. We investigate the transport of ionic species at cell/encapsulant interfaces, demonstrating that POE limits both sodium and silver ion migration compared with EVA. Changes to the chemical structures of the encapsulants at the cell/encapsulant interfaces demonstrate that both POE and EVA are more susceptible to degradation in modules with a transparent backsheet than in the G/G configuration. Adhesion testing reveals that POE and EVA have comparable critical debond energies after the DH exposures regardless of system bias polarity. The results of this study indicate that the interfacial degradation mechanisms of G/G appear to be similar to those of conventional glass/backsheet modules. For emerging materials, our results demonstrate that POE offers advantages over EVA but that transparent backsheets may accelerate encapsulant degradation due to increased moisture ingress when compared with the G/G structure.

## Graphical abstract

G/G modules with POE were found to be more durable than those with EVA or transparent backsheet. The high resistivity POE inhibits sodium migration through the interfaces under negative and silver migration under positive bias, decreasing risk of PIDs and transmittance loss as compared to EVA. Interfacial adhesion strength reduced for both EVA and POE encapsulants resulting in similar risks of delamination under the damp heat conditions.

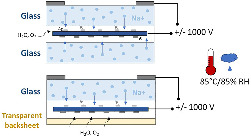

## 1 INTRODUCTION

The reliability of bifacial photovoltaic (PV) modules has been gaining attention in recent years due to increasing demand for larger system power outputs. The market share of bifacial modules is expected to increase from 30% in 2020 to about 80% within the next 10 years.^1^ Bifacial solar cells can achieve greater power densities by leveraging rear‐side illumination, but they require transparent module back coverings, such as glass or transparent polymer backsheets. Glass/glass (G/G) packaging has been implemented in thin‐film and building‐integrated PV technologies but is less common in silicon (Si) PV modules. G/G modules are more rigid and less permeable to moisture and oxygen than conventional glass/backsheet (G/B) modules due to the glass covering on both sides. Historical field data from early‐generation G/G Si PV modules encapsulated with poly(ethylene‐co‐vinyl acetate) (EVA) suggest that these modules suffer from more severe power losses than their G/B counterparts.^2^ The degradation modes typically observed in these historic modules include delamination, encapsulant discoloration, busbar and junction box corrosion, and glass cracking.^3^

Although historic G/G modules suffered from some critical issues, modern G/G modules are emerging with better packaging and more robust mounting hardware. Alternative encapsulants to EVA have been implemented to mitigate the degradation of G/G PV modules and maintain long‐term field durability. Polyolefin elastomer (POE) is a promising candidate to replace EVA due to the lack of the degradation byproduct, acetic acid. Entrapment of acetic acid in the semi‐impermeable G/G module structure is the hypothesized cause of increased degradation rates and metallization corrosion.^2^, ^3^ However, there is some difficulty in comparing classes of polymer materials, as these materials are formulated with various types and amounts of additives to enhance performance and cost for different applications. Generally, POEs also have higher volume resistivities and lower water vapor transmission rates (WVTRs) than EVAs and are thus likely to limit ionic and moisture transport through the encapsulant.^4^, ^5^, ^6^ POEs thus reduce the risk of potential‐induced degradation (PID) mechanisms in Si modules.^7^ Finally, POEs typically have higher melting points than EVAs, which may be mechanically advantageous in G/G modules that operate at slightly higher temperatures.^8^

Glass/transparent backsheet (G/TB) PV modules are also emerging as an alternative to G/G modules for enabling bifacial PV. G/TB technology promises less weight, lower module operating temperatures, and established fabrication processes. The long‐term durability of transparent backsheets is still under investigation; currently, there are limited field and accelerated weathering data.^9^, ^10^, ^11^ In particular, maintaining high transmittance and mechanical properties over long periods of time remains a challenge in certain backsheet formulations.^10^, ^11^

Thus, the durability of the module materials in the G/G and G/TB configurations requires further analysis. Previous work on conventional G/B with EVA has shown bias‐induced failures such as loss of adhesion and sodium ion migration under negative bias^12^ and silver migration under positive bias.^13^ The question of how these degradation modes and failure mechanisms translate to the G/G configuration, given that the moisture transport is expected to be severely limited, remains unanswered. With the quickly increasing popularity of G/G modules, it is of high importance to understand the distinct long‐term reliability risks of both G/G and related bifacial module packaging materials, such as transparent backsheets and POE.

Our previous studies used nondestructive characterization to compare the performance degradation pathways in bifacial Si PV mini‐modules in G/G and G/TB configurations with either EVA or POE encapsulation.^14^ We subjected the mini‐modules to damp heat (DH, 85°C/85% relative humidity) with no bias (DH) and with positive (DH+) and negative (DH−) system voltage biases of 1000 V. POE‐encapsulated modules experienced the least degradation in all test conditions, with less than 5% power loss compared with a maximum of 35% for EVA‐containing modules. We observed PID involving shunting (PID‐s) in the EVA‐containing modules under DH− for both G/G and G/TB. PID‐s is typically associated with the migration of positively charged ions (Na+) from glass to the cell interface under the negative bias, which decorates Si stacking faults and causes shunts across the p‐n junction.^15^ We found that a separate mechanism, PID with polarization (PID‐p), affected the rear side of the passivation stack for only the G/G EVA module under DH‐. We found this mechanism to be fully recoverable. Polarization of the rear surface in bifacial p‐type PERC cells has been attributed to the attraction of positive charges to the aluminum oxide/silicon nitride (AlO_x_/SiN_x_) stack, which decreases the field‐effect passivation and leads to increased surface recombination.^16^ Lastly, we observed local corrosion of the metallization (PID‐c and/or moisture‐induced corrosion) in all the G/TB modules, as well as in the EVA‐containing G/G modules. We attributed the corrosion in the G/TB modules to the greater moisture permeability of backsheets compared with glass. We observed the greatest power loss in the EVA G/G modules under DH+. Although this type of degradation is not well understood, it has been associated with various phenomena, including negative charge migration to the AlO_x_/SiN_x_ passivation layer, gridline corrosion, silver migration into the encapsulant, electrochemical degradation of SiN_x_, and local Si corrosion, which leads to delamination of the passivation and impurity accumulation.^13^, ^17^, ^18^

The aim of this work is to investigate the chemical and mechanical degradation of the materials and interfaces in G/G modules. Because PID is often attributed to ion migration, we begin with surface‐sensitive X‐ray photoelectron spectroscopy (XPS) to characterize the ionic migration and changes in chemical bonding at the interfaces between the cell, encapsulant, and glass. We then further elucidate the changes in the chemical structures of the encapsulants at the cell/encapsulant interfaces using Fourier transform infrared spectroscopy (FTIR). Adhesion strength is known to be impacted by changes to the interfacial chemistry, so we measure interfacial adhesion to evaluate the risk of long‐term packaging issues such as delamination. The results from this study highlight the importance of encapsulant choice and demonstrate the chemical and mechanical degradation mechanisms of G/G module packages as compared wih conventional G/B. This provides guidance on the material and packaging designs that may limit degradation and extend PV module lifetimes.

## 2 EXPERIMENTAL METHODS

### 2.1 Sample preparation

Single‐cell mini‐module samples measuring 200 × 200 mm were prepared using bifacial monocrystalline p‐type passivated emitter and rear contact (p‐PERC) cells measuring 156 ⨯ 156 mm, commercial EVA‐ or POE‐based encapsulants, and 3.2‐mm‐thick tempered low‐iron textured Solite (AGC) glass on the front side. Half of the module set used glass as the back cover, and half used a transparent backsheet. Silver gridlines were used on the front side of the cell, and aluminum was used on the rear side. Mini‐modules were subjected to accelerated stress testing consisting of 85°C, 85% RH and zero (DH), positive (DH+), or negative (DH−) system voltage of 1000 V for 1000 h. This is a significantly longer exposure than the 96‐h PID test specified in the International Electrotechnical Commission 61215‐2 Module Qualification Test 21^19^ and was chosen to detect long‐term durability issues for the G/G and G/TB package beyond initial PID susceptibility. One module of each type was set aside and stored at room temperature to serve as a control sample. After stress testing, modules were characterized nondestructively using current–voltage, external quantum efficiency, photoluminescence, electroluminescence, and dark lock‐in thermography. More details on sample preparation, stress testing, and module characterization are provided in Sulas‐Kern et al.^14^ Table 1 summarizes the test samples forming a total of 16 mini‐modules.

**TABLE 1:** Mini‐modules and stress conditions

Module geometry	Encapsulant type	Stress test conditions (1000 h)			
G/G	EVA	Reference (no applied stress)	DH (85°C/85% RH, no bias)	DH+ (85°C/85% RH, 1000 V)	DH− (85°C/85% RH, −1000 V)
	POE				
G/TB	EVA				
	POE				

Abbreviations: DH, damp heat; EVA, poly(ethylene‐co‐vinyl acetate); G/G; glass/glass; G/TB, glass/transparent backsheet; POE, polyolefin elastomer.

### 2.2 Chemical analysis

We performed XPS on samples cored from the mini‐modules. Encapsulant and cell pieces were extracted from the mini‐modules by coring through the glass/encapsulant/cell stack using a 1/2‐inch diameter diamond drill bit while constantly lubricating the cored area using RedLube polishing lubricant (Allied High Tech Products, Inc.). Samples were extracted by attaching a stainless steel metal stub to the cover glass in the cored area, then pulling and twisting the stub until the stack was separated at the weaker interface.^20^, ^21^ The XPS spectra were collected in a PHI VersaProbe III system (ɸ ULVAC‐PHI, Inc.) with a hemispherical analyzer. The X‐ray beam spot size was 100–200 μm in diameter and was sensitive to surface analysis, with a penetration depth of 3–7 nm. A neutralizer was used to minimize any charging effects on the cell and encapsulant surfaces. X‐ray‐induced secondary electron imaging of the cell surface allowed us to locate the narrow gridline position and then simultaneously analyze the cell gridline region and a region on the anti‐reflection coating (ARC) away from the gridline. For this, we narrowed the X‐ray beam diameter down to 100 μm and collected high‐resolution XPS spectra at a pass energy of 55 eV and an energy resolution of 0.1 eV. The data were acquired with multiple sweeps to increase the signal‐to‐noise ratio. The binding energy (BE) scale calibration was done using an adventitious carbon (C 1s) peak at 284.8 eV. We resolved overlapping peaks into individual components by fitting Gaussian–Lorentzian functions and obtained the atomic composition from integrated peak intensities using the CasaXPS software package (Casa Software Ltd.). The data presented below are from a single point on the sample, and multiple points and cored areas from each module were analyzed to confirm consistency.

We performed FTIR on the freshly fractured surfaces of the adhesion test coupons (described in Section 2.3) using the attenuated total reflection accessory of a Bruker ALPHA II compact spectrophotometer. Scans were conducted from 4000 to 400 cm^−1^. We measured several points in the region of interest and then averaged and normalized the spectra after using the automatic baseline correction available in the Bruker OPUS software package. Intensities were normalized using the 2850 cm^−1^ peak, corresponding to the C–H stretching of the ethylene group.

### 2.3 Adhesion testing

Adhesion testing of full G/G modules or mini‐modules is not possible due to the thickness and stiffness of the glass covers, whether tempered or untempered. Fundamentally, adhesion testing requires controlled and measurable removal of outer layers, which is prohibited by the double‐glass module construction. Thus, we fabricated separate adhesion test coupons using the same materials as the mini‐modules in Section 2.1, complete with bussing to enable voltage bias, but with a few distinctions to enable the adhesion testing. First, we replaced the back glass with 4.5″ × 6.25″ microscope cover glass that was less than 250 μm thick.^22^ Cells were halved via laser scribing such that the cover glass was larger than the cell area. Next, an LPS dry film polytetrafluoroethylene (PTFE) lubricant was used to create a pre‐crack at the rear cell/back encapsulant interface. Other interfaces were prepared with pre‐cracks, but in these tests, the crack would travel to and then propagate along the cell/encapsulant interface, indicating that this is the interface of interest. Titanium cantilever beams were adhered to the cover glass with an epoxy. The loading tab was then screwed to the beam. The cover glass was scribed with a diamond tip, and a razor was used to create the cantilever sections. More details can be found in the Supporting Information.

The adhesion test coupons were examined with the cantilever beam technique before and after the stress conditions listed in Table 1, using a miniature tension/compression load frame (DTS Delaminator, Menlo Park) at a constant displacement rate of 10 μm/s. The critical debond energy ( $G_{I_{c}}$) was calculated using the modified compliance calibration method detailed in ASTM D5528‐13.^23^ In this method, the relationship between the beam compliance, C, and the crack length, a, is found by loading and unloading the beam with various crack lengths. The beam compliance is proportional to the cube of the crack length, $a^{3}$, so $G_{I_{c}}$ is calculated as follows:

(1) $$G_{I_{c}}=\frac{3P^{2}C^{2/3}}{2\mathrm{Abh}},$$

where P is the maximum load, b is the beam width, h is the beam height, and A is the slope of a fitted line for a versus C13. More details and alternative methods are available in ASTM D5528‐13.^23^

## 3 RESULTS AND DISCUSSION

### 3.1 Interfacial chemistry

G/EVA/G modules under DH− suffered degradation from a combination of PID mechanisms, including shunting, polarization, and corrosion. G/EVA/TB modules under DH− similarly suffered from PID‐s and PID‐c. However, in the G/POE/G modules, these mechanisms were suppressed. Both PID‐s and PID‐p have been attributed to ion migration between the glass and the cell. Although studies have shown that PID‐s is specifically caused by sodium migration that eventually creates shunt pathways within the Si cell,^15^ PID‐p is a less well‐understood mechanism.^17^ We observed signatures of PID‐p at the center of the cell causing enhanced carrier recombination at the rear side, suggesting a temporary positive charge accumulation at the rear cell surface.^14^ To date, the species responsible for PID‐p has not been identified, but early studies suggest it may not be caused by sodium migration.

Because PID‐s and PID‐p appear to be driven by ion migration, we turned to chemical analysis with XPS, which is surface‐sensitive and useful for chemical characterization at interfaces. To determine the location of any chemical species, we focused our analysis on the encapsulant/cell interface and measured in two distinct regions on the cell surface—above gridlines and between gridlines (identified herein as the ARC region).

### 3.2 Chemical analysis: Negative bias

**FIGURE 1:**
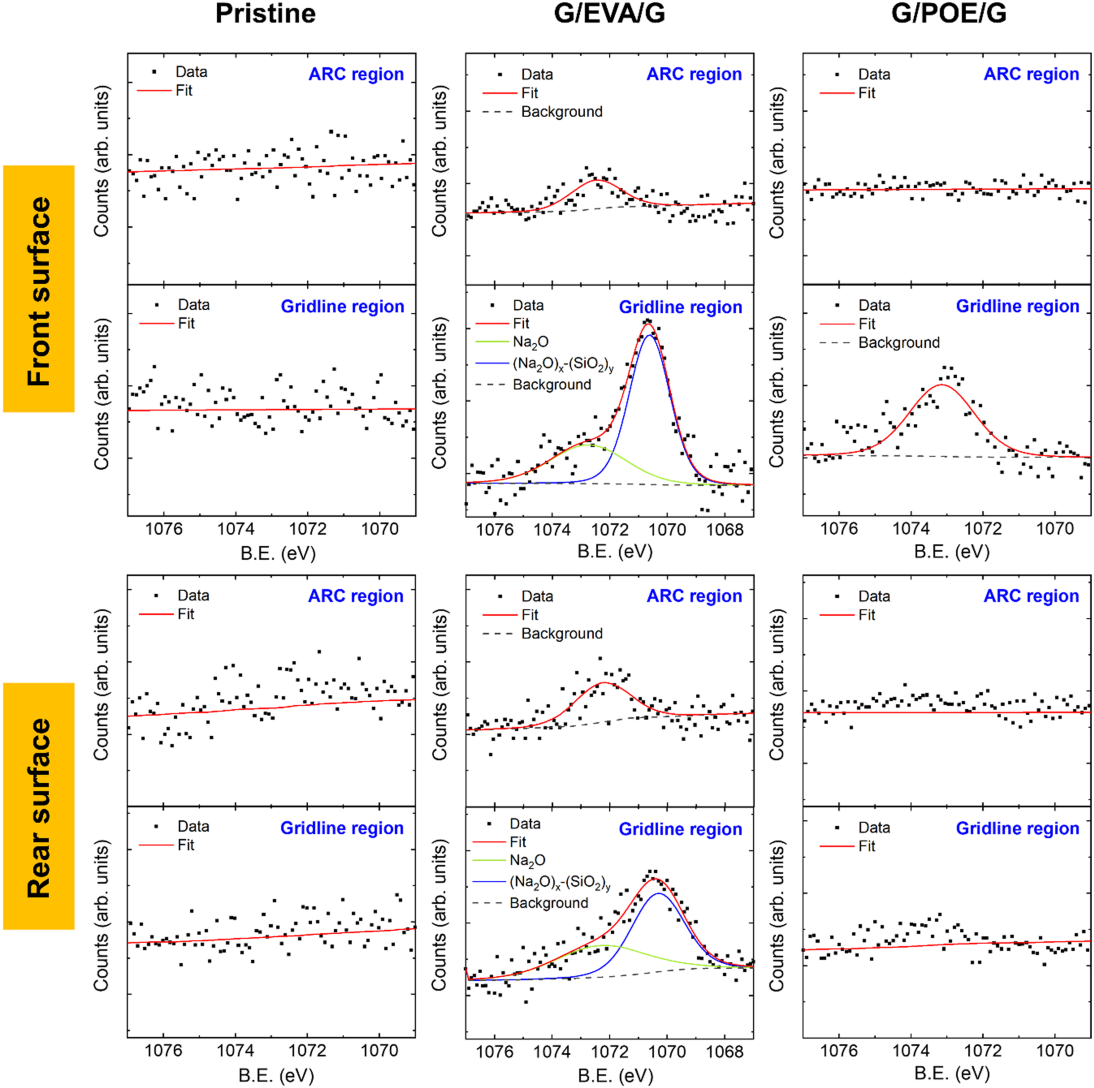
X‐ray photoelectron spectroscopy (XPS) Na 1s signal at the front and rear interfaces of the pristine Si cell and the cells extracted from G/EVA/G and G/POE/G mini‐modules aged under DH−

Figure 1 shows cell XPS results from the Na 1s spectra. As in previous work on G/G and conventional G/B modules,^12^ we see preferential migration of sodium to the gridlines on the front side of the cell. In the gridline region of the G/EVA/G module, the Na 1s signal was fit to two peaks. The peak fit BEs indicate the presence of sodium oxide (Na_2_O) and sodium silicate (Na_2_O)_x_ (SiO_2_)_y._ ^24^ Sodium silicate has been linked to adhesion loss in previous glass/white backsheet studies.^12^ Although this is not a direct measure of the sodium associated with PID‐s, the presence of sodium in the EVA sample suggests ion migration from glass to the cell surface, allowing for eventual migration into the cell and subsequent shunting. In comparison, the sodium signature was greatly suppressed in the POE samples, suggesting decreased ion migration in the POE. In addition, sodium silicate was not detected on the cell surface of the G/POE/G module. POEs are known to have lower moisture permeabilities and higher volume resistivities than EVA,^14^, ^25^ and the results presented here indicate that the POE used in this study also impedes ion migration more than EVA. For the particular EVA and POE encapsulants used in this study, the volume resistivity of POE was measured to be 1000× greater compared with EVA, and the volume resistivity of EVA decreased upon moisture saturation.^14^ Decreased ion migration in POEs implies lower risk of power loss via PID‐s. The sodium signal also shifted to a higher BE in the POE samples, indicating that sodium may interact with the POE differently than with the EVA.

For both encapsulants, more sodium was detected on the front side of the cell than on the rear side. These samples were fabricated with an Al tape on the front side of the mini‐module, which may have enhanced the electric field on that side in comparison with the weaker floating potential on the rear.^26^

### 3.3 Chemical analysis: Positive bias

G/EVA/G modules suffered from severe (39 rel.% PCE loss) rear‐side degradation under DH+. Only negligible degradation was observed for other module configurations (G/EVA/TB and G/POE/G) with the same applied stress.

**FIGURE 2:**
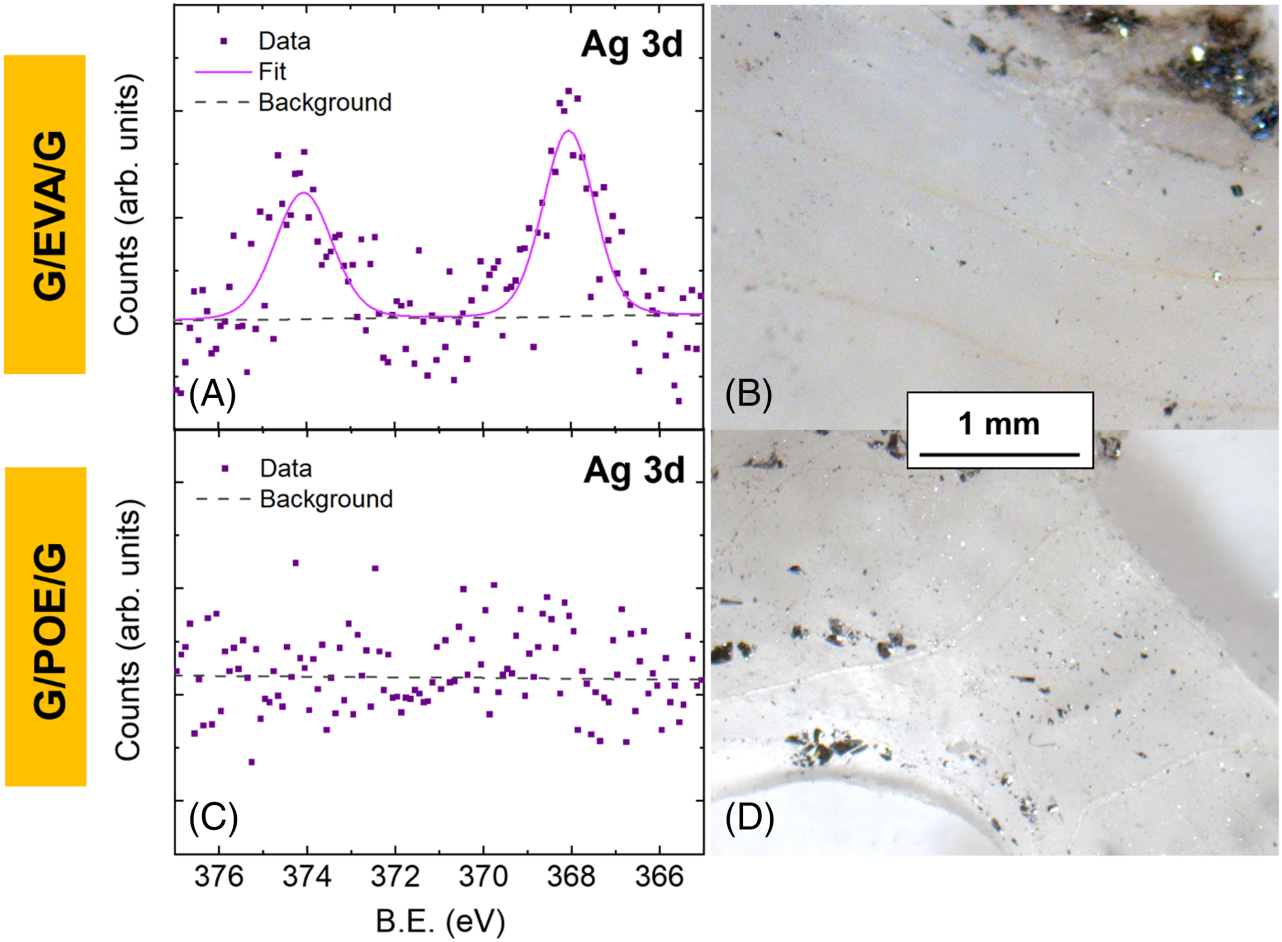
(A) X‐ray photoelectron spectroscopy (XPS) Ag 3d spectra on the surface of the front poly(ethylene‐co‐vinyl acetate) (EVA) encapsulant over the cell gridline and (B) corresponding microscope image showing discolored lines of front gridline/encapsulant interface in G/EVA/G under DH+. (C) XPS Ag 3d spectra collected from the front gridline region of POE encapsulant and (D) corresponding microscope image from G/POE/G under DH+

We examined cored sections of the encapsulants using XPS. As shown in Figure 2, silver was present in the encapsulant for the G/EVA/G DH+ sample, and the encapsulant was discolored over the gridlines, similar to what has been observed in G/B modules with EVA.^13^ This discoloration was not observed in the POE sample nor in the samples under negative bias. This demonstrates that POE not only limits sodium migration under negative bias but also limits silver migration under positive bias. The silver migration may decrease the encapsulant transmittance via discoloration, subsequently reducing *J*_*sc*_, and we suggest that it may also contribute to a reduction in the encapsulant‐gridline adhesion.

**FIGURE 3:**
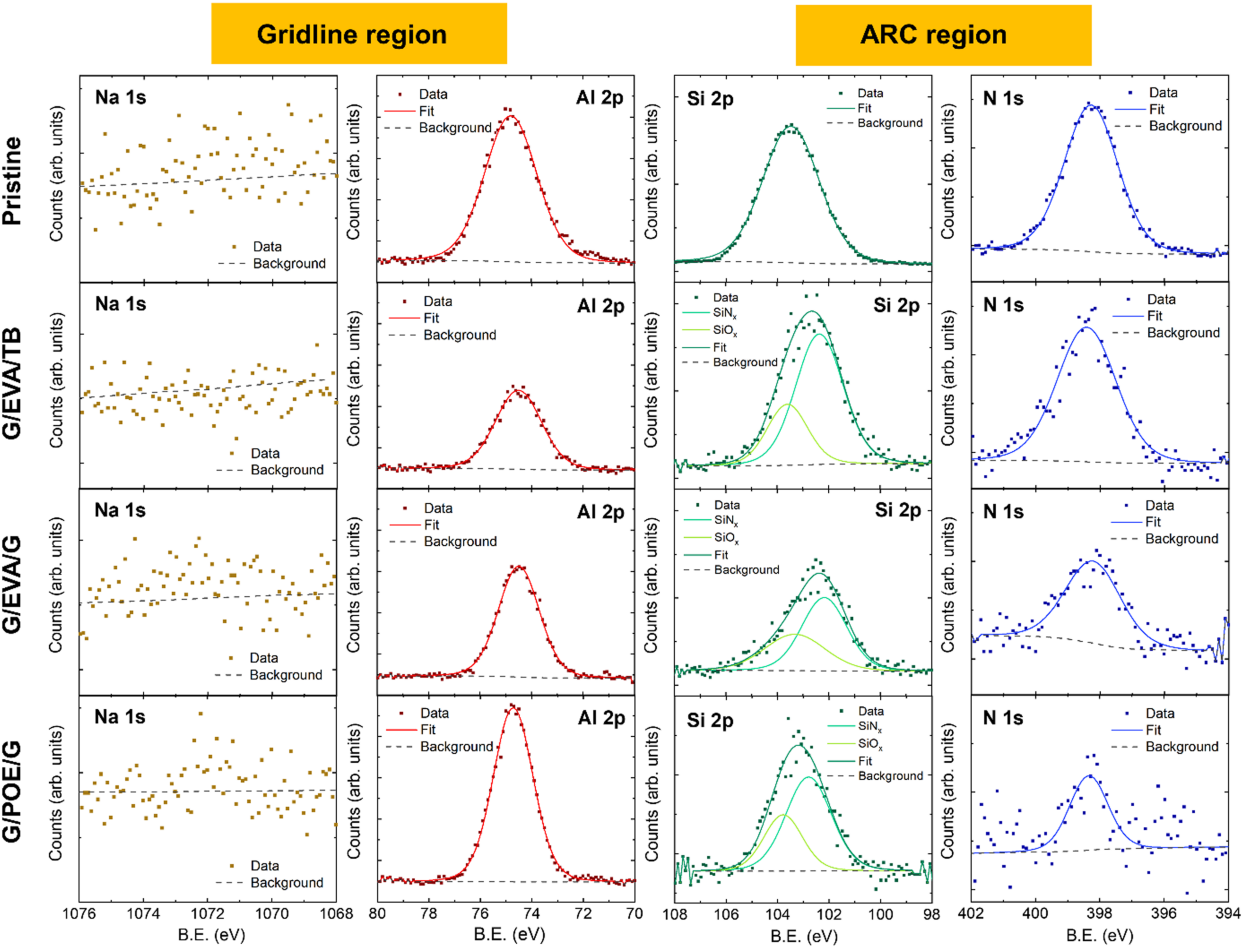
Elemental composition at the rear cell/encapsulant interface for three different module configurations under DH+ compared with the pristine unencapsulated cell. *Y*‐axes are normalized to have the same number of counts, except in the case of the Si and N signals for the pristine cell, which had much higher counts overall

Figure 3 shows XPS chemical analysis on the rear interface, comparing possible elemental migration and gridline corrosion in different packaging configurations. As expected, no significant sodium signals were present in the positive bias case. The aluminum signal indicated aluminum oxide, which is expected for the surfaces of the rear contacts due to the high reactivity of aluminum. The aluminum peak positions and shapes are consistent across all the DH+ samples and the pristine cell, indicating no apparent gridline corrosion. Additionally, Al migration to the encapsulant was not detected. The nitrogen (N) and Si signals were also consistent across the samples, with no major peak shifts or distortions.

These results indicate that the significant rear‐side power loss under DH+ is not due to elemental migration or gridline corrosion. Although no chemical changes were detected for the ARC, we suggest that the power loss may be due to other related mechanisms, such as impurity accumulation at buried interfaces (SiN_x_/AlO_x_ or AlO_x_/Si) or thinning of the rear‐side passivation leading to increased recombination. These mechanisms require further investigation for the case of G/G modules using EVA as an encapsulant.

### 3.4 Encapsulant chemistry

**FIGURE 4:**
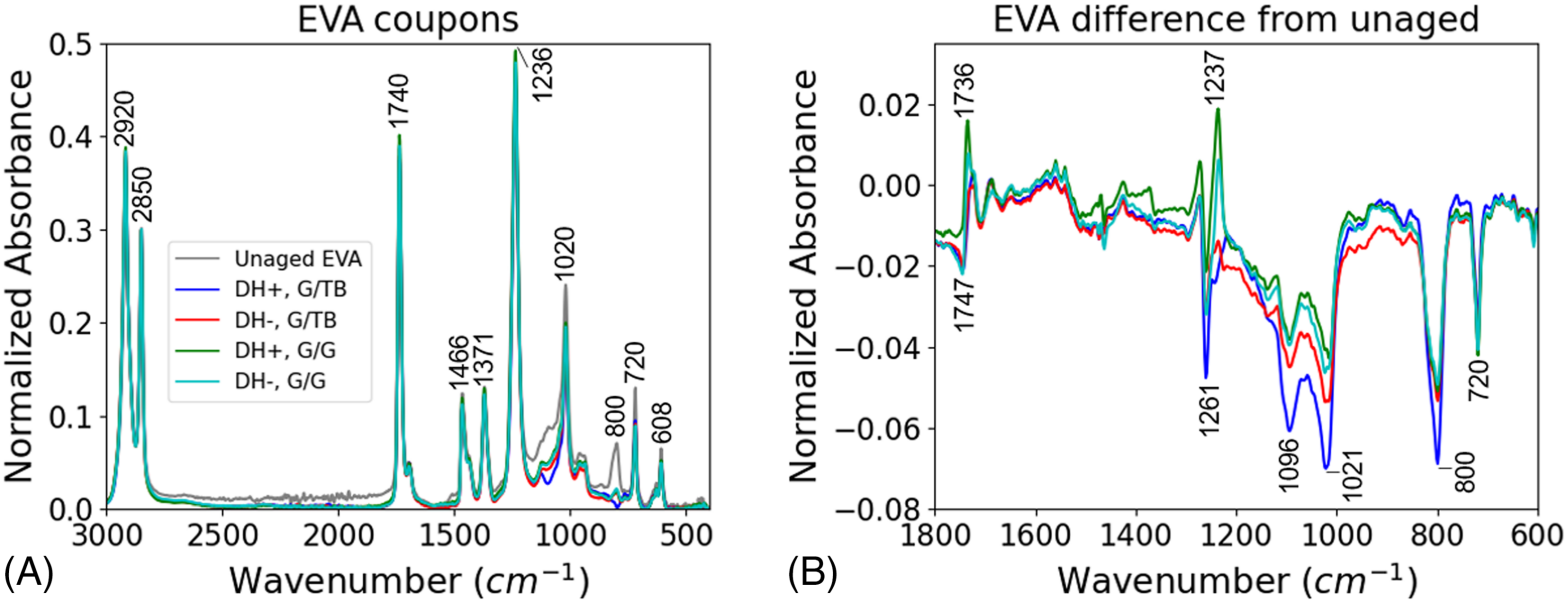
(A) Fourier transform infrared spectroscopy (FTIR) of poly(ethylene‐co‐vinyl acetate) (EVA) adhesion coupons and (B) spectra differences from the unaged sample

To further understand the chemical effects of aging in G/G packaging, we investigated the chemical structures of the encapsulants using FTIR. The FTIR results show that the stress conditions degraded the chemical structures of the EVA in comparison with the unaged samples (Figure 4). For the EVA, the 1260, 1100, 1020, and 800 cm^−1^ peaks demonstrated lowered intensities with aging, consistent with other results.^27^, ^28^ The decreased 1260 and 1020 cm^−1^ peaks correspond to C–O–C functional group typically present in vinyl acetate moieties and common additives such as cross‐linking agent or silane.^27^, ^29^ The decreased 1100 and 800 cm^−1^ peaks (vibration of Si–O) correspond to decreased silane bonding at the rear cell/encapsulant interface, which has been linked to adhesion strength.^30^, ^31^ Deacetylation is unlikely taking place in these samples as minor to no changes were observed in carbonyl and hydroxyl regions of the spectra. The polarity of the bias did not have an immediate effect on the encapsulant chemical structures.

**FIGURE 5:**
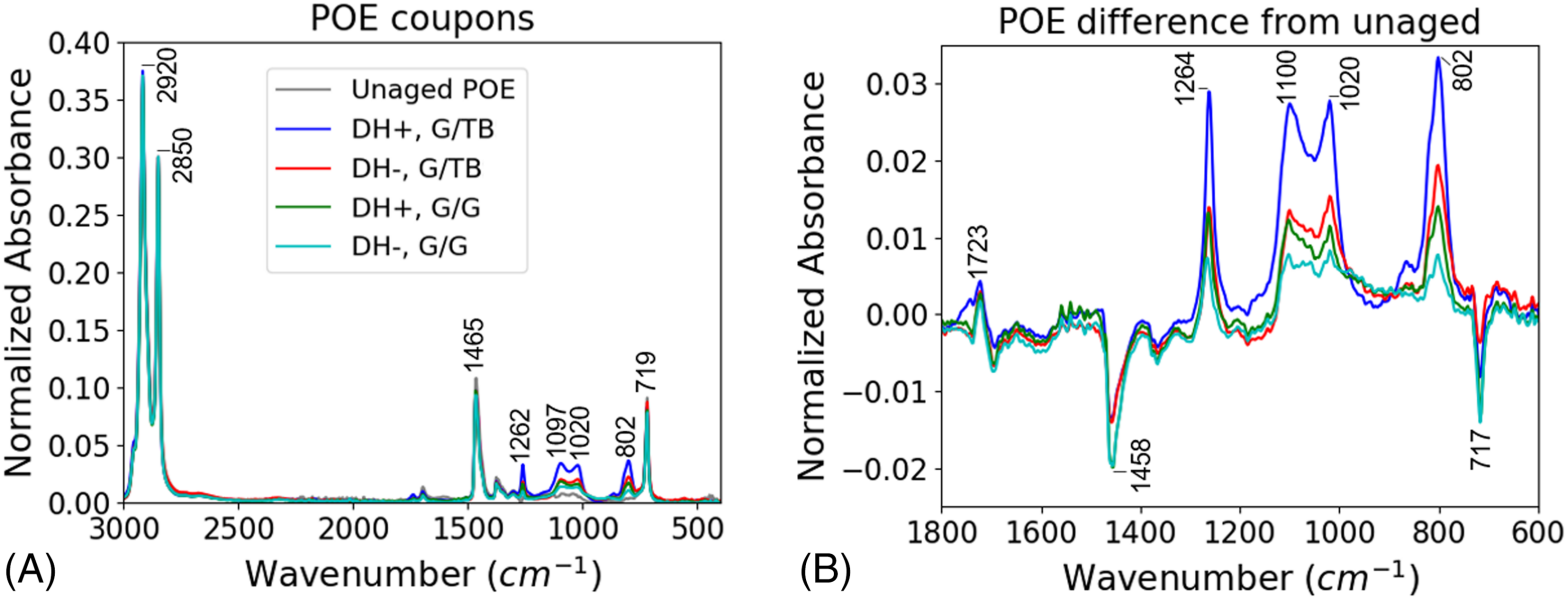
(A) Fourier transform infrared spectroscopy (FTIR) of polyolefin elastomer (POE) adhesion coupons and (B) spectra differences from the unaged sample

**FIGURE 6:**
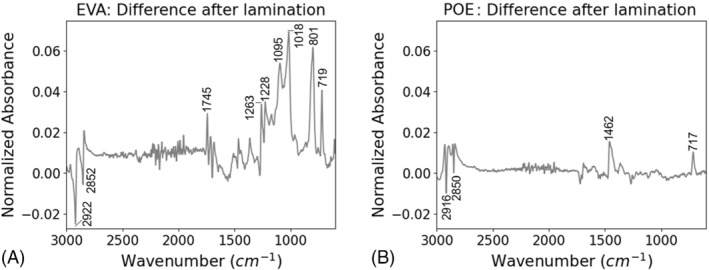
Fourier transform infrared (FTIR) spectra of fresh encapsulant subtracted from unaged but packaged encapsulant for (A) poly(ethylene‐co‐vinyl acetate) (EVA) and (b) polyolefin elastomer (POE). The difference after lamination is much greater for the EVA

The chemical structure of the POE was also affected by the DH aging without sensitivity to bias polarity, as shown in Figure 5. In contrast to the EVA results, the POE demonstrated increases in the peak intensities at 1260, 1100, 1020, and 800 cm^−1^. POE lacks the vinyl acetate unit. The presence of these peaks in both EVA and POE confirms their origin from common encapsulant additives and/or silane. The inverse behavior of these peaks suggests that the additives and/or silane were altered in a different way by aging in POE than in EVA. To understand this phenomenon, Figure 6 compares the FTIR spectra of unlaminated encapsulant and encapsulant from an unaged sample. Whereas the EVA structure was affected immediately by lamination via crosslinking and silane bonding, the POE did not show immediate effects. This presents two possibilities. First, the measurements were taken at the cell/encapsulant interface, and these results may indicate additive blooming, which is a phase segregation and migration of the additives/silane to this interface, in the POE during aging.^32^ Second, given that POE is known to have higher volume resistance and lower moisture permeability,^14^ the reactions that occur quickly in EVA may be occurring more slowly throughout aging in POE.

Although EVA and POE appear to degrade differently, both encapsulants showed greater chemical effects for the G/TB construction than for the G/G construction. This indicates that in DH, the greater moisture ingress of the polymer backsheet has a larger impact on the encapsulant structures than the restricted moisture egress of the G/G construction.

### 3.5 Adhesion testing

In addition to the chemical degradation discussed above, one additional degradation mode in PV modules is delamination. As noted above, early‐generation G/G modules suffered from extensive delamination failure, and adhesion was reduced by the interfacial chemistry. Here, to determine the risk of delamination under different bias conditions and with different encapsulants, we measured adhesion of the glass/encapsulant/cell interfaces.

**FIGURE 7:**
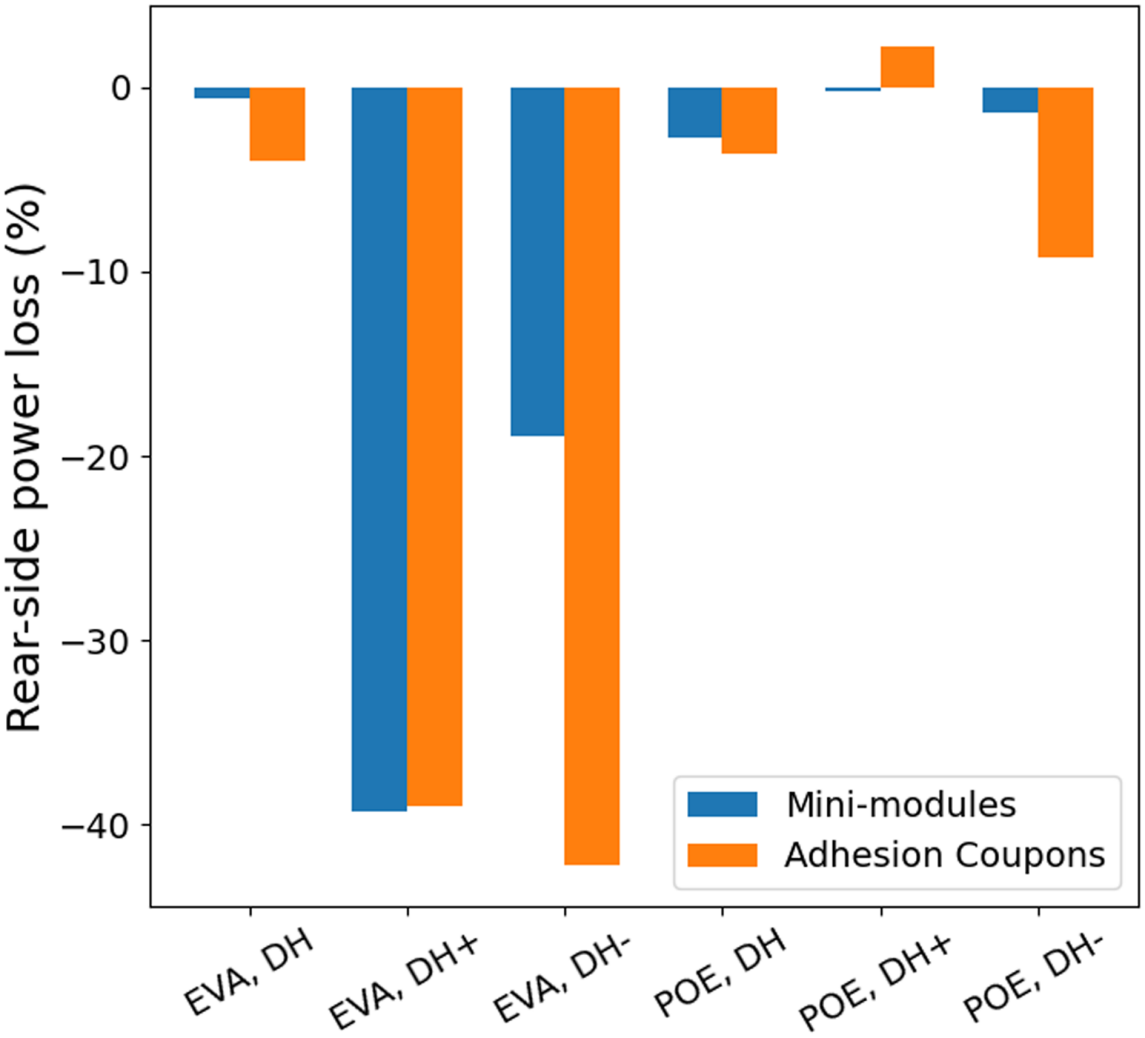
Percent rear‐side power loss comparison for the glass/glass (G/G) mini‐modules and the G/G adhesion test coupons under the three stress conditions. Similar trends are noted despite differences in construction

To assess the usefulness of the adhesion test coupons, we compared the rear‐side power loss of the adhesion test coupons to that of the mini‐modules in Figure 7. For both, test coupons and mini‐modules, the power was obtained by measuring the rear‐side IV curves at standard test conditions (STCs), as described in our previous work.^14^ The overall power loss trends are similar, with the adhesion test coupons degrading more rapidly. The adhesion coupons may have greater moisture ingress than the mini‐modules due to the higher edge‐to‐volume ratio, the pre‐crack at one interface, and cracks in the cover glass away from the cell. While this method of measuring interfacial adhesion losses may not be directly comparable with the fielded modules, the similarities in the rear‐side power loss trends validate the use of these test coupons for the adhesion measurements and provide a useful comparison between the EVA and POE encapsulant adhesion strength before and after aging.

**FIGURE 8:**
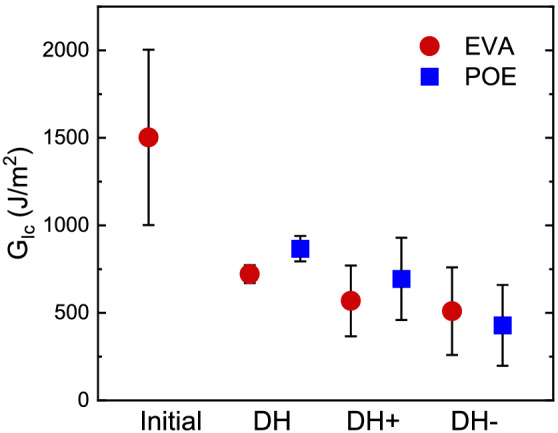
Debond energies for the glass/glass (G/G) adhesion test coupons before and after exposure. The mean value was obtained from 4 or more measurements per sample, and error bars represent 95% confidence intervals. The initial polyolefin elastomer (POE) value could not be measured due to the high strength of the unaged interface

Adhesion results for rear cell/encapsulant interfaces are presented in Figure 8. The samples were designed to closely match module construction, with the tradeoff that the additional material layers and bussing introduced greater variability in the measurements. Initial adhesion measurements of the unaged POE samples could not be attained due to the high strength of the unaged interfaces. The EVA samples demonstrated good initial adhesion. All three of the stress conditions caused significant losses in adhesion strength. The EVA and POE adhesion test coupons may have had different initial debond energies, but their values were similar (within the error of the measurement) after aging. Although the average debond energy was found to be generally higher after DH than after DH+/− for both EVA and POE, this result does not appear to be statistically significant.

The results indicate that DH exposure decreases adhesive strength. EVA and POE demonstrated similar debond energies following the exposures, indicating that neither is advantaged in terms of delamination resistance under DH. The debond energies after aging were higher than the delamination threshold of 200 J/m^2^ found by Bosco et al.,^33^ which was developed for fielded G/B modules. In the case of G/G modules, the stresses acting upon the interfaces are expected to be somewhat higher,^8^, ^34^ requiring a greater interfacial debond energy to prevent delamination. Ultimately, these results represent only DH and DH/voltage conditions, so sequential accelerated test sequences involving mechanical stressors and thermal cycling are recommended to further characterize the risk of delamination in fielded G/G modules.

## 4 CONCLUSIONS

In this study, we assessed the interfacial phenomena that impact the durability of G/G module packaging with EVA‐ and POE‐based encapsulants as compared with transparent backsheet packages. The high resistivity and impermeability of the POE used here were found to hinder both sodium and silver ion migration, decreasing the risk of PID‐s and transmittance loss as compared with the EVA. Although the FTIR spectra for the POE were shown to transform with aging, these chemical changes did not appear to impact the POE's resistance to ion migration. There was some evidence that the lower permeability of the POE led to additive blooming at the POE interfaces, but both the EVA and the POE demonstrated similar risks of delamination under the damp heat conditions, regardless of the voltage bias. This study demonstrated that, regardless of the encapsulant used, the interfacial failure mechanisms present in G/G, such as ion migration and adhesion loss, are similar to those observed in conventional G/B modules. However, measurements of the encapsulant chemical structures revealed greater changes for the G/TB modules, indicating that G/G packages may restrict the kinetics of deleterious chemical phenomena through lower moisture ingress, giving G/G some advantages over G/B packages.

## Supporting information

Supporting Information

## Data Availability

The data that support the findings of this study are available from the corresponding author upon reasonable request.
