# Supplementary material for: Chemical and mechanical interfacial degradation in bifacial glass/glass and glass/transparent backsheet photovoltaic modules
Source: Prog Photovolt. 2022 Jun 29;30(12):1423–32. doi: 10.1002/pip.3602 (PMC9796659; doi:10.1002/pip.3602)
Supplement: Supplementary file 1 — Supporting Information [file PIP-30-1423-s001.pdf]

Supporting Information:

Chemical and mechanical interfacial degradation in bifacial glass/glass and glass/transparent backsheet photovoltaic modules

Laura Spinella*, Soňa Uličná+, Archana Sinha+, Dana B. Sulas-Kern*, Michael Owen-Bellini*, Steve Johnston*, and Laura T. Schelhas*

**National Renewable Energy Laboratory, Golden, CO*
+*SLAC National Accelerator Laboratory, Menlo Park, CA*

Here we show schematics of the adhesion test coupons and the single-cell mini-modules. The key difference is demonstrated in Fig. S1, namely, that the adhesion test coupons substitute the back glass for a thin cover glass, i.e., a large microscope slide.

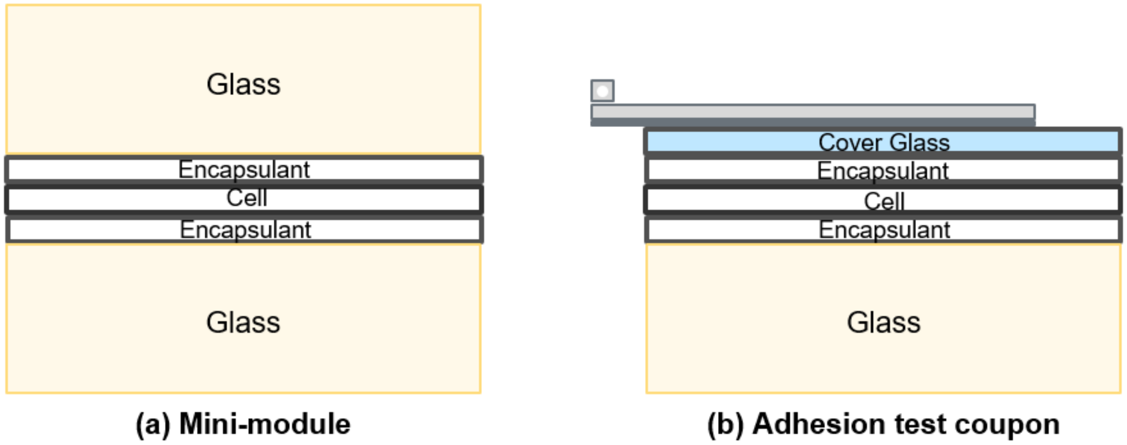

Fig. S1. Cross-sectional schematics of the (a) mini-modules and (b) adhesion test coupons. Beam with loading tab are attached with epoxy directly following exposures.

Figs. S2 and S3 demonstrate the differences in construction of the single-cell mini-module and the adhesion test coupon. Fig. S2 shows a mini-module in the typical glass/encapsulant/cell/encapsulant/glass configuration as well as with ribbons and busbars. Fig. S3 shows the adhesion test coupon configuration. Due to size constraints for the thin coverslip glass, we used half-cells. We applied PTFE to the rear cell to create a pre-crack at the rear cell/encapsulant interface in the region indicated in Fig. S3.

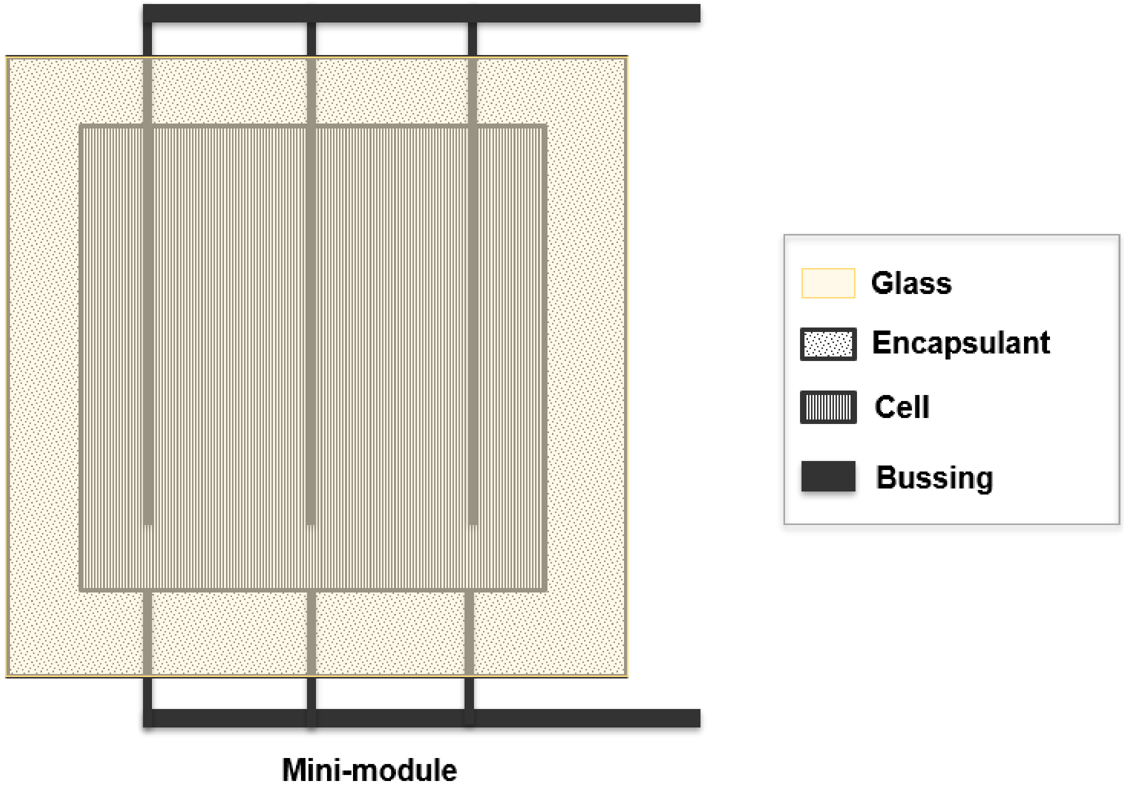

Fig. S2. Aerial view of single-cell mini-module.

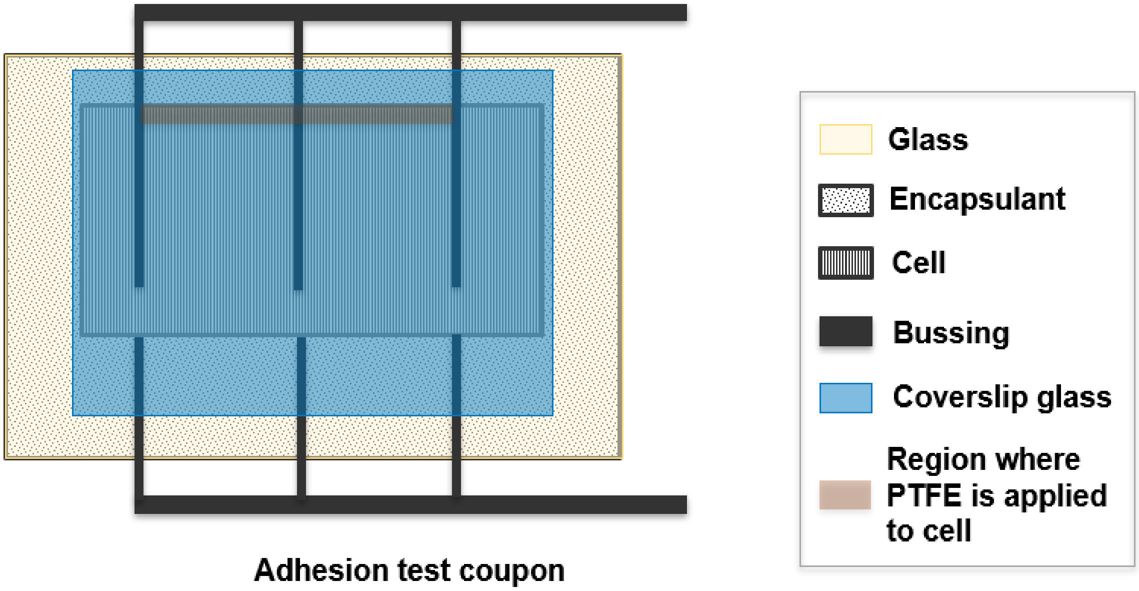

Fig. S3. Aerial view of adhesion test coupon.
